# Exogenous Lipoid Pneumonia Complicated by Mineral Oil Aspiration in a Patient With Chronic Constipation: A Case Report and Review

**DOI:** 10.7759/cureus.9294

**Published:** 2020-07-20

**Authors:** Hafiz Muhammad Jeelani, Muhammad Mubbashir Sheikh, Belaal Sheikh, Hafiz Mahboob, Anchit Bharat

**Affiliations:** 1 Internal Medicine, Rosalind Franklin University of Medicine and Science, McHenry, USA; 2 Oncology, Northwestern University Feinberg School of Medicine, Chicago, USA; 3 Internal Medicine, Rosalind Franklin University of Medicine and Science, North Chicago, USA; 4 Internal Medicine, Chicago Medical School, North Chicago, USA; 5 Pulmonary and Critical Care Medicine, University of Nevada Las Vegas School of Medicine, Las Vegas, USA; 6 Internal Medicine, Indiana University Health Ball Memorial Hospital, Muncie, USA

**Keywords:** lipoid pneumonia, mineral oil, constipation, bal lavage, macrophages

## Abstract

Exogenous lipoid pneumonia is a rare and frequently misdiagnosed lung disease. It occurs as an inflammatory reaction secondary to either aspiration or inhalation of lipids. Our patient had a history significant for recurrent pneumonia and the use of mineral oil for chronic constipation. A chest computed tomography showed multifocal consolidative opacities with areas of low attenuation, highly suspicious of exogenous lipid pneumonia. The diagnosis was confirmed with combined bronchoalveolar lavage and transbronchial lung biopsy that showed lipid-laden macrophages consistent with exogenous lipoid pneumonia. After thorough medication review, apart from mineral oil, no other contributing factors were found. A diagnosis of exogenous lipoid pneumonia associated with the use of mineral oil made and successfully managed by stopping the offending agent and supportive antibiotics.

## Introduction

Lipoid pneumonia has been identified as a non-infectious cause of recurrent aspiration pneumonia in 1-2.5% cases. Histologically, it is characterized by the lipid-laden macrophages in the alveoli [1]. Exogenous lipoid pneumonia (ELP) is a subtype that occurs as an inflammatory reaction to the exogenous lipids of mineral, animal, or vegetable origin, either via inhalation or aspiration [2]. The other two etiologies of lipoid pneumonia include endogenous (intrinsic aspiration via endobronchial obstruction of lipids within alveoli, chronic infections, and lipid storage disorders) and idiopathic [3]. Association with laxatives use has been reported as an important and most frequent risk factor for ELP [2]. Here, we describe an unusual and rare case of exogenous lipoid pneumonia secondary to aspiration of mineral oil used as a laxative for chronic constipation. Diagnosis requires a high degree of suspicion and can be confirmed by lung biopsy. This article addresses the significance of a comprehensive review of over-the-counter medications used for underlying conditions that can benefit in timely diagnosis and preventing future debilitations.

## Case presentation

**Figure 1:**
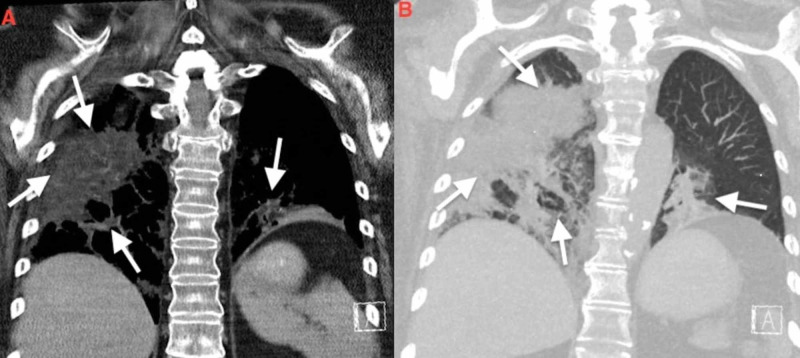
CT Chest showing multifocal, bilateral, consolidative opacities with areas of low density ranging from -91 HU to -47 HU involving bilateral lower lobe and right upper lobe (white arrows in panel A and B) CT: Computed tomography

**Figure 2:**
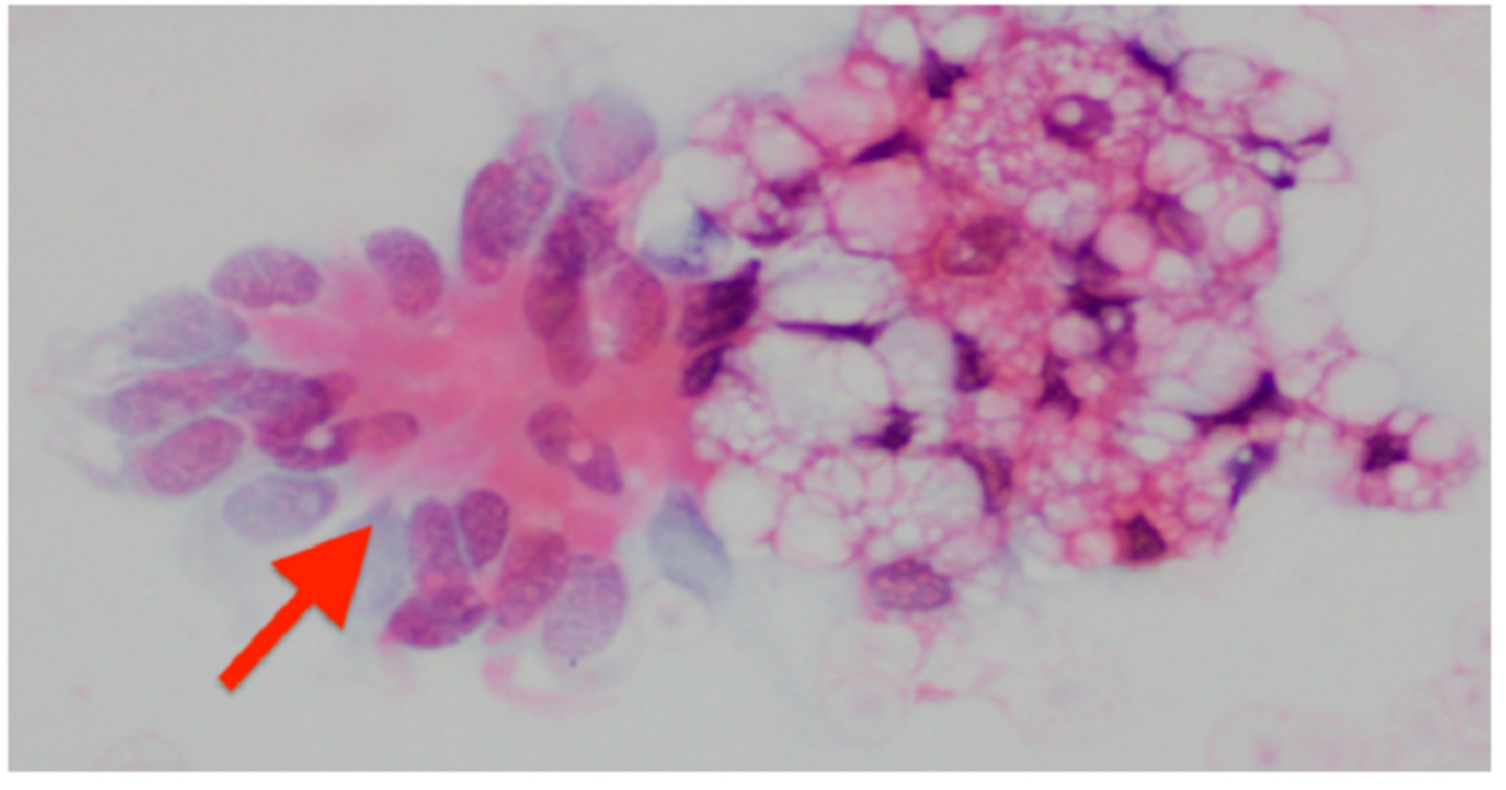
Cytological findings of the BAL showing lipid-laden macrophages (red arrow) BAL: bronchoalveolar lavage

**Figure 3:**
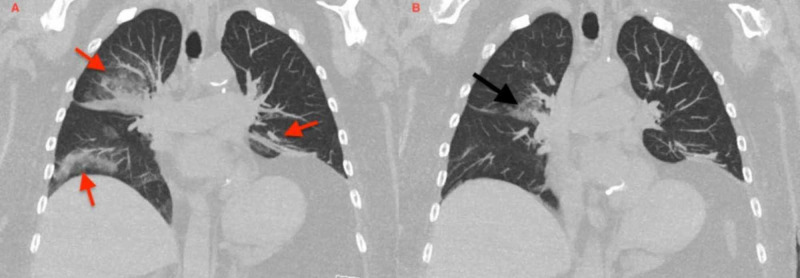
Follow-up CT Chest at three months (panel A) showed partial improvement of bilateral consolidative opacities (red arrows). Follow-up CT at six months (panel B) showing complete resolution of RUL, improved bilateral lower lobes airspace opacities, and a patchy consolidative opacity in right middle-lower lobe (black arrow) secondary to resolving ELP CT: computed tomography, RUL: right upper lobe, ELP: exogenous lipoid pneumonia

A 66-year-old Caucasian female with a past medical history significant for multiple sclerosis, chronic constipation, and recurrent pneumonia presented to the emergency department (ED) with sudden onset of dyspnea. Prior to admission, she completed the course of broad-spectrum antibiotics for her third episode of pneumonia. Focused physical examination revealed a low-grade temperature of 99°F, oxygen saturation of 86% on room air, and coarse breath sounds on auscultation. Laboratory investigation were significant for elevated white blood cell (WBC) count of 15.9 cells/mm^3^ (normal range: 4-11 cells/mm^3^) with 89% of neutrophils (normal range: 40%-75%). Comprehensive metabolic panel and coagulation profile were within a normal range. Initially, she was stabilized with appropriate oxygenation. Later, a chest computed tomography (CT) scan was administered. In comparison to previous imaging, CT showed extensive multifocal consolidative opacities with a bilateral lower lobe predominance and involvement of the right upper lobe, and areas of low attenuation, highly suggestive of lipoid pneumonia (Figure 1). Transbronchial biopsy with bronchoalveolar lavage (BAL) and a re-start of broad-spectrum antibiotics was recommended by pulmonary evaluation. Cytopathology of the BAL revealed reactive bronchial cells and lipid-laden macrophages consistent with the diagnosis of lipoid pneumonia (Figure 2). A focused review of the medication list revealed the repeated use of mineral oil for chronic constipation. Mineral oil was stopped, and the patient was continued on supportive antibiotics therapy. Her symptoms improved, and the patient was discharged to a skilled nursing facility. Follow up CT scans at three months and six months showed significant resolution of upper lobe infiltrates, and improvement in lower lobe opacities with no exudative lesions or lymphadenopathy (Figure 3).

## Discussion

Chronic constipation is a common debilitating gastrointestinal disorder in the elderly. For rapid relief, available remedial options include stool softeners, fiber supplements, osmotic and stimulant laxatives. Of all the options, mineral oil is preferred laxative by the physicians in the elderly. It is a tasteless, odorless, and indigestible liquid acts by slowing down the colonic absorption of water and softens the stool. Owing to these peculiar characteristics, it may suppress the normal gag and cough reflex, and mucociliary functions, thus facilitating aspiration [4]. Once aspirated into the lungs, mineral oil is phagocytosed by alveolar macrophages resulting in chronic alveolar and interstitial inflammation [5].

Exogenous lipoid pneumonia (ELP) is classically linked to aspiration of lipids, specifically to mineral oil, reported earlier as the most common cause, and described in this case as well. Predisposing conditions that increase the risk of ELP include extremes of age, anatomical and functional abnormalities of pharynx/esophagus, and neuromuscular disorders [6-9]. However, 25% of ELP cases have been reported in healthy individuals without any underlying conditions [2]. The clinical presentation of ELP is subtle and is dependent on the patient’s age, volume, and duration of exposure to the offending agent. The most common reasons for consultation include exertional dyspnea, hypoxia, and recurrent pneumonia. A physical examination can be normal or demonstrate dullness to percussion, wheezing, crackles, or rhonchi on auscultation [2-4].

The diagnosis is challenging due to variable clinical and radiological findings. The common differentials that mimic ELP include infectious pneumonia, mycobacterial tuberculosis, Wegener’s granulomatosis, and bronchogenic carcinoma, and should be kept in mind [10]. History consistent with exposure to the offending agents, classical radiological features, and BAL analysis aid in diagnosing ELP and differentiating from the mentioned differentials. CT findings highly suggestive for ELP include consolidation with areas of fat attenuation, i.e., negative attenuation values between -150 HU to -30 HU, ground-glass opacities, crazy paving pattern, airspace nodules, interlobular septal thickening, interstitial fibrosis, and mass-like lesions [11]. CT in our patient revealed multifocal, consolidative opacities of low density ranging from -91 HU to -47 HU involving bilateral lower lobe and right upper lobe shown in (Figure 1). The macroscopic finding of whitish or turbid fluid with fat globules on the fluid surface are characteristic findings on BAL, as seen in this case as well (Figure 2) [12].

Treatment is not well defined for ELP. After confirmation via biopsy and high-resolution computed tomography (HRCT), discontinuing the offending agent is the mainstay and sufficient treatment [13]. The resolution of symptoms and radiographic abnormalities is usually apparent within months. Early recognition and management are recommended for ELP associated complications (Table 1) [14-16]. In our case, discontinuing the mineral oil and supportive management along with antibiotics showed significant improvement. Other treatment options include broad-spectrum antibiotics to prevent superimposed infections (Table 1). Therapeutic lung lavage and glucocorticoids have also shown beneficial effects in a limited number of cases [12,17,18].

**Table 1:** Complications associated with ELP *: common complications, **: rare Complications, ELP: exogenous lipoid pneumonia

Common and rare complications
Superimposed infections (Nocarida and Mycobacterial)*
Bronchiectasis*
Pulmonary Fibrosis*
Respiratory failure**
Cor-pulmonale**
Bronchogenic carcinoma**

## Conclusions

In summary, exogenous lipoid pneumonia should be considered a possible diagnosis in case of any non-resolving pneumonia. Elderly patients presenting with recurrent pneumonia should always be asked about the history of constipation and mineral oil use. Identification and discontinuation of the offending agent is the key to management. Bronchoscopy with BAL should be done for early diagnosis and confirmation whenever this disease is suspected. If untreated, exogenous lipoid pneumonia can be complicated by superimposed infections, respiratory failure, or bronchogenic carcinoma.
